# Sleep‐related safety behaviours predict insomnia symptoms 1 year later in a sample of university students

**DOI:** 10.1111/jsr.14381

**Published:** 2024-10-17

**Authors:** Jaap Lancee, Jan Henk Kamphuis

**Affiliations:** ^1^ Department of Clinical Psychology University of Amsterdam Amsterdam The Netherlands

**Keywords:** cognitive factors, insomnia, safety behaviours

## Abstract

Several studies have demonstrated the relevance of cognitive factors in the development of insomnia complaints, but very few have investigated how these factors influence the development of insomnia complaints over time. In this study we set out to investigate key factors associated with present insomnia severity and the development of insomnia complaints over time. We employed a two‐wave longitudinal design where we measured insomnia severity, pre‐sleep arousal, dysfunctional beliefs about sleep, sleep‐related worry and safety‐behaviours in a sample of students at baseline and 1 year later. At baseline, 353 respondents filled in the questionnaires and 79 completed these a year later. In the cross‐sectional analyses, pre‐sleep arousal and sleep‐related worry were unique contributors to insomnia severity. Using baseline data to predict insomnia severity 1 year later, only sleep‐related safety emerged as a predictor. These findings suggest that sleep‐related worry and pre‐sleep arousal are the primary factors influencing current severity. In terms of development and/or persistence, sleep safety may constitute a potentially underestimated factor.

## 1 INTRODUCTION

Chronic insomnia is a prevalent and very debilitating disorder. The current gold‐standard for insomnia is cognitive behaviour therapy (CBT‐I; Riemann et al., 2023). Even though CBT‐I is an effective treatment format, there is still a large group of patients that does not remit after this treatment (Edinger et al., 2021). It is therefore of major importance to further improve treatment for insomnia. A first crucial step in improving treatments is to understand which factors are important contributors to the severity of the symptoms and to the maintenance of these symptoms over time.

An influential model on the formation and maintenance of insomnia is the cognitive model of insomnia (Harvey, 2002). This model suggests that repetitive thoughts about potential sleep deficits and the repercussions of poor sleep contribute to heightened arousal and distress. Consequently, this heightened state leads to focused attention and heightened vigilance towards sleep‐related threats. These processes, in turn, foster an exaggerated perception of the impact of sleep difficulties, further worsening sleep and daytime functioning. Dysfunctional beliefs and safety behaviours perpetuate and intensify these processes, thereby playing a crucial role in maintaining insomnia. These cognitive processes are not only central in Harvey's model but also in most other models of insomnia (Tang et al., 2023).

Empirically, several authors indeed demonstrated that these cognitive factors are related to insomnia severity (Harvey, 2002; Hiller et al., 2015; Norell‐Clarke et al., 2014, 2021; Vand et al., 2014) and are related to treatment progress (Lancee et al., 2022; Sunnhed et al., 2022). To date, however, there are only a few studies that have investigated the combined influence of factors of the cognitive model in the maintenance of insomnia over time. Specifically, in a large epidemiological study, Norell‐Clarke et al. (2014, 2021) investigated the full cognitive model of insomnia (except for the impact of distorted perception of sleep deficit). They observed that worry, dysfunctional beliefs, somatic arousal, selective attention and monitoring, and safety behaviours were all related to current insomnia complaints, and that a decline in selective attention and safety behaviours was related to remission from insomnia (Norell‐Clarke et al., 2014). Moreover, they observed that baseline safety behaviours and somatic arousal were associated with the development of insomnia over time (Norell‐Clarke et al., 2021).

The present paper contributes to the literature on the combined influence of cognitive factors over time. Specifically, we set out to investigate how pre‐sleep arousal, dysfunctional beliefs about sleep, sleep‐related worry and safety‐behaviours are: (1) associated with insomnia complaints; and (2) if any of these factors predict insomnia complaints 1 year later. To this aim we administered questionnaires on dysfunctional beliefs, safety behaviours, pre‐sleep arousal, sleep‐related worry and insomnia complaints to university students, and repeated the same measurements 1 year later.

## 2 METHODS

### 2.1 Participants

Participants were psychology students from the University of Amsterdam, the Netherlands: 401 students started the first wave and 353 completed all questionnaires. In this sample, the mean age was 20.1 years (SD = 1.65) and 241 (68.3%) were female. In the second wave, 79 students (22.3%) filled out the questionnaires again and 72 fully completed both measures. Of the 79 students, the mean age at baseline was 20.1 years (SD = 1.81) and 63 (79.7%) were female. At baseline, the groups completing and not completing the second wave did not differ on most variables (*p* > 0.05). The only exception was that the group not completing the second wave scored higher on sleep safety behaviours (*M* = 32.87; SD = 16.55) than the group that did complete the second wave (*M* = 26.17; SD = 16.30, *t*_374_ = 3.19, *p* = 0.02; see Table 1 for descriptives).

**TABLE 1:** Descriptives.

	*n*	*M*	SD
Baseline			
Sleep worry (APSQ)	380	21.49	8.58
Dysfunctional beliefs (DBAS)	379	4.53	1.51
Pre‐sleep arousal (PSAS)	377	31.06	9.89
Sleep safety behaviours (SRBQ)	376	31.52	16.69
Insomnia severity (ISI)	377	7.01	4.93
Perseverative thinking (PTQ)	382	28.07	12.08
1 year later			
Sleep worry (APSQ)—1 year	79	19.96	8.08
Dysfunctional beliefs (DBAS)—1 year	79	3.44	1.48
Pre‐sleep arousal (PSAS)—1 year	79	30.72	10.13
Sleep safety behaviours (SRBQ)—1 year	79	27.00	14.66
Insomnia severity (ISI)—1 year	79	6.85	4.85
Perseverative thinking (PTQ)—1 year	79	23.01	12.19

APSQ, Anxiety and Preoccupation about Sleep Questionnaire; DBAS, Dysfunctional Belief and Attitudes About Sleep; ISI, Insomnia Severity Index; PSAS, Pre‐Sleep Arousal Scale; PTQ, Perseverative Thinking Questionnaire; SRBQ, Sleep‐Related Behaviours Questionnaire.

### 2.2 Measurements

In line with the suggestions of Hiller et al. (2015), we used the following questionnaires at both time‐points. (1) Insomnia severity was measured with the seven‐item Insomnia Severity Index (ISI; Bastien et al., 2001), with higher scores indicating more complaints (range: 0–28). (2) Dysfunctional beliefs were assessed with the Dutch translation of the 16‐item Dysfunctional Belief and Attitudes about Sleep scale (DBAS; Morin et al., 2007). The sum of the DBAS score is averaged so that the total score ranges from 0 (no dysfunctional beliefs) to 10 (severe dysfunctional beliefs). (3) Sleep‐related arousal was measured with the 16‐item Pre‐sleep Arousal Scale (PSAS), with higher scores indicating more arousal (range 16–80; Nicassio et al., 1985). (4) Sleep‐related worry was measured with the 10‐item Anxiety and Preoccupation about Sleep Questionnaire (APSQ), with higher scores indicating more worry (range 10–50; Jansson‐Frojmark et al., 2011). (5) Sleep‐related safety‐behaviours were measured with the 32‐item Sleep‐Related Behaviours Questionnaire (SRBQ). The total score ranges from 0 (no safety behaviours) to 128 (severe safety behaviours; Ree & Harvey, 2004). Perseverative thinking was measured with the 15‐item Perseverative Thinking Questionnaire (PTQ), with higher scores indicating more perseverative thinking (Ehring et al., 2011).

### 2.3 Procedure

All data were collected at the Psychology Department of the University of Amsterdam as part of the first‐year psychology program in 2013. All students were informed about the general nature of the questionnaires, and gave informed consent with the possibility to retract their data until 3 weeks after the questionnaires. Every student who completed questionnaires in 2013 was approached again in 2014. They were requested to complete the same set of sleep‐related questionnaires and to consent to the merging of their 2014 and 2013 data. Students received study credits or a 5 Euro monetary reward for filling out these questionnaires. The internal Ethical Review Board of the University of Amsterdam approved this procedure (2014‐CP‐3828).

### 2.4 Statistical analysis

We calculated zero‐order correlations between all relevant variables. Next, we performed forced‐entry hierarchical regression analyses with the ISI as the dependent variable. First we performed two cross‐sectional regression analyses on each wave separately, with ISI as the dependent variable, and PSAS, DBAS, APSQ and sleep safety as the independent variables. To test for maintaining factors, we ran a regression analysis with the second wave ISI score (1 year) as the dependent variable, and first wave variables as the independent variables. In the first block we added baseline ISI. In the second block we added baseline PSAS, DBAS, APSQ and sleep safety. All analyses were carried out with SPSS (IBM, version 28.0.1.0).

Assumptions were checked for the residuals, and we detected one multivariate outlier on the multiple regression analysis of the first wave. This participant did not complete the second wave. We ran the analyses with and without this case included in our data set, which did not change the results of our analyses. We decided to retain this case in the analyses and report the analysis without the outlier in the supplemental file.

## 3 RESULTS

See Table 1 for mean scores on all the included variables and Table 2 for the zero‐order correlations. All baseline variables showed significant correlations with insomnia severity 1 year later (*p* < 0.05).

**TABLE 2:** Zero‐order correlations between baseline and 1‐year follow‐up variables.

	APSQ	DBAS	PSAS	SRBQ	ISI	APSQ 1y	DBAS 1y	PSAS 1y	SRBQ 1y
Baseline									
Sleep worry (APSQ)									
Dysfunctional beliefs (DBAS)	0.67**								
Pre‐sleep arousal (PSAS)	0.58**	0.45**							
Sleep safety behaviours (SRBQ)	0.60**	0.60**	0.570**						
Insomnia severity (ISI)	0.67**	0.49**	0.62**	0.51**					
1 year later									
Sleep worry (APSQ)—1 year	0.49**	0.46**	0.22	0.50**	0.38**				
Dysfunctional beliefs (DBAS)—1 year	0.49**	0.58**	0.21	0.41**	0.37**	0.80**			
Pre‐sleep arousal (PSAS)—1 year	0.46**	0.35**	0.59**	0.47**	0.50**	0.55**	0.53**		
Sleep safety behaviours (SRBQ)—1 year	0.60**	0.53**	0.52**	0.74**	0.47**	0.65**	0.62**	0.67**	
Insomnia severity (ISI)—1 year	0.38**	0.29*	0.44**	0.44**	0.55**	0.66**	0.53**	0.73**	0.63**

Baseline correlations are based on *n* = 360–377. One‐year correlations are based on *n* = 76–79.

APSQ, Anxiety and Preoccupation about Sleep Questionnaire; DBAS, Dysfunctional Belief and Attitudes About Sleep; ISI, Insomnia Severity Index; PSAS, Pre‐Sleep Arousal Scale; SRBQ, Sleep‐Related Behaviours Questionnaire.

* *p* < 0.05;

** *p* < 0.01.

### 3.1 Cross‐sectional analyses

At the first time‐point, APSQ and DBAS showed significant relationships to insomnia severity (*F*_4,348_ = 103.04, *p* < 0.001, $R_{\mathrm{adj}}^{2}$ = 53.7%). At the second time‐point, 1 year later, the APSQ and DBAS again showed significant relationships to insomnia severity (*F*_4,74_ = 33.01, *p* < 0.001, $R_{\mathrm{adj}}^{2}$ = 62.1%; Table 3).

### 3.2 Analyses predicting insomnia severity 1 year later

In the first block, insomnia severity at baseline predicted insomnia severity 1 year later (*F*_1,69_ = 31.24, *p* < 0.001, $R_{\mathrm{adj}}^{2}$= 30.2%). In the second block, the other baseline variables were added. Now insomnia severity *and* safety behaviours predicted insomnia severity 1 year later (*F*_5,65_ = 7.45, *p* < 0.001, $R_{\mathrm{adj}}^{2}$ = 31.5%). None of the other variables was significantly related to insomnia severity a year later. We also performed the regression analyses with somatic and cognitive pre‐sleep arousal as separate variables. In this analysis again only safety behaviours and insomnia severity were significant predictors (*F*_6,64_ = 6.11, *p* < 0.001, $R_{\mathrm{adj}}^{2}$ = 31.0%; Table 4).

**TABLE 3:** Regression coefficients for the cross‐sectional analyses predicting insomnia severity (ISI).

Baseline	*B*	SE	*β*	*t*
(Constant)	−4.68	0.69		−6.79***
Sleep worry (APSQ)	0.26	0.03	0.45	8.16***
Dysfunctional beliefs (DBAS)	0.12	0.17	0.04	0.73
Pre‐sleep arousal (PSAS)	0.17	0.02	0.35	7.41***
Sleep safety behaviours (SRBQ)	0.00	0.01	0.01	0.85
$R_{\mathrm{adj}}^{2}$	53.7%			

1 year later	*B*	SE	*β*	*t*
(Constant)	−4.99	1.16		−4.29***
Sleep worry (APSQ)	0.26	0.07	0.43	3.47**
Dysfunctional beliefs (DBAS)	−0.50	0.39	−0.15	−1.26
Pre‐sleep arousal (PSAS)	0.24	0.05	0.51	5.28***
Sleep safety behaviours (SRBQ)	0.04	0.04	0.11	1.00
$R_{\mathrm{adj}}^{2}$	62.1%			

Baseline is based on *n* = 353 and 1‐year follow‐up on *n* = 79.

APSQ, Anxiety and Preoccupation about Sleep Questionnaire; DBAS, Dysfunctional Belief and Attitudes About Sleep; PSAS, Pre‐Sleep Arousal Scale; SRBQ, Sleep‐Related Behaviours Questionnaire.

** *p* < 0.01.

*** *p* < 0.001.

**TABLE 4:** Regression coefficients predicting 1‐year follow‐up insomnia severity with baseline measures.

Step 1	*B*	SE	*β*	*t*
(Constant)	3.40	0.80		4.25***
Insomnia severity	0.56	0.10	0.56	5.59***
$R_{\mathrm{adj}}^{2}$	30.2%			

Step 2	*B*	SE	*β*	*t*
(Constant)	4.56	2.31		1.98
Sleep worry (APSQ)	−0.01	0.11	−0.01	−0.06
Dysfunctional beliefs (DBAS)	−0.39	0.51	−0.12	−0.77
Pre‐sleep arousal (PSAS)	−0.06	0.10	−0.10	−0.62
Sleep safety behaviours (SRBQ)	0.11	0.05	0.37	2.21*
Insomnia severity	0.48	0.14	0.48	3.44**
$R_{\mathrm{adj}}^{2}$	31.5%			

Regression model is based on *n* = 71.

APSQ, Anxiety and Preoccupation about Sleep Questionnaire; DBAS, Dysfunctional Belief and Attitudes About Sleep; PSAS, Pre‐Sleep Arousal Scale; SRBQ, Sleep‐Related Behaviours Questionnaire.

* *p* < 0.05.

** *p* < 0.01.

*** *p* < 0.001.

## 4 DISCUSSION

In this study we investigated how dysfunctional beliefs about sleep, sleep‐related worry, pre‐sleep arousal and sleep‐related safety behaviours related to current insomnia severity and insomnia severity over time. Cross‐sectionally we observed that, in line with earlier research (Norell‐Clarke et al., 2014), all variables showed strong correlations with insomnia severity. When added into one model, only pre‐sleep arousal and sleep‐related worry remained significant associations with insomnia severity. This does not fully align with the observations of Norell‐Clarke et al. (2014), where safety behaviours and dysfunctional beliefs remained significant contributors in the final model. It does seem to fit into the cognitive model where cognitive activity is the entry point of the model that directly taps into arousal and distress (Harvey, 2002). In the model, dysfunctional beliefs and safety behaviours predominantly play a role in sustaining insomnia and may not be exclusive factors in determining current severity levels.

In the analyses concerning the longitudinal data, all baseline variables individually correlated with insomnia severity levels 1 year later. However, when added into the multiple regression model, only safety behaviours showed a unique relationship with insomnia severity. This relationship even survived controlling for baseline insomnia severity. This partly aligns with a previous study where safety behaviours and somatic arousal were unique contributors to insomnia incidence 6 months later (Norell‐Clarke et al., 2021).

Before further appraising this result, we think it is important to mention several limitations to this study. First, this study was conducted in a student sample with relatively low insomnia levels, and findings may thus not generalise to people with insomnia disorder. Second, we did not measure selective attention (e.g. with the Sleep Associated Monitoring Index; Semler & Harvey, 2004) and distorted perception (e.g. with objective measurements), which are also important factors in the cognitive model of insomnia. Third, this study was carried out in a sample of predominantly female psychology students, which limits the generalisability of our findings. Fourth, we had no a‐priori hypothesis that safety behaviours would be the most important factor, the findings are therefore exploratory in nature. Fifth, attention checks were not used, and we could therefore not ascertain whether respondents were (dis‐)engaged. Sixth and finally, only a small percentage of respondents also completed the 1‐year measurement, which may have biased the results.

Keeping these limitations in mind, we think it is noteworthy that different variables are associated with concurrent insomnia severity than with its development/maintenance over time. Consistent with the cognitive model of insomnia (Harvey, 2002), we found that safety behaviour is indeed an important factor contributing to the maintenance of insomnia (although it is unclear why dysfunctional beliefs seem of lesser importance). Effects for safety behaviour are also in line with the Attention‐Intention‐Effort model where the similar construct of sleep effort plays an important role (Espie et al., 2006). For example, in a student population, these behavioural sleep efforts/safety behaviours may include maladaptive behaviour like extending morning risetimes in order to catch up on sleep. This may lead to negative consequences such as missing lectures, which may cascade into other problems. Moreover, as Espie et al. (2006) argue, their effort to obtain more sleep may in fact lead to reduced sleep efficiency.

These data suggest a central role of safety behaviours in the maintenance of insomnia symptoms. Hence, specifically targeting and comprehensively fading safety behaviours – as also observed in the context of panic disorder (Kamphuis & Telch, 1998) may produce a more complete and enduring effect in terms of prevention and/or treatment. Clearly, the present data only allow for conjectures, and we recommend that they be put to the empirical test in high‐quality insomnia treatment trials.

## AUTHOR CONTRIBUTIONS

**Jaap Lancee:** Conceptualization; data curation; formal analysis; writing – original draft; methodology; investigation; supervision; project administration; writing – review and editing. **Jan Henk Kamphuis:** Conceptualization; writing – review and editing; methodology; investigation; supervision.

## CONFLICT OF INTEREST STATEMENT

The authors have no conflict of interest for this study.

## Supporting information


DATA S1.

## Data Availability

The data that support the findings of this study are available from the corresponding author upon reasonable request.
