# Supplementary material for: Sleep‐related safety behaviours predict insomnia symptoms 1 year later in a sample of university students
Source: J Sleep Res. 2024 Oct 17;34(3):e14381. doi: 10.1111/jsr.14381 (PMC12069750; doi:10.1111/jsr.14381)
Supplement: Supplementary file 1 — DATA S1. [file JSR-34-e14381-s001.docx]

**Authors**

Jaap Lancee & Jan Henk Kamphuis

**Title**

Sleep-related safety behaviors predict insomnia symptoms one year later in a sample of university students.

**Online supplemental file**

Assumptions were checked for the residuals, and we detected one multivariate outlier on the multiple regression analysis of the first wave. In the main document we presented the analyses with this outlier included, here we present the regression model without the outlier included.

| **Supplemental Table S1 - Regression coefficients for the cross-sectional analysis predicting insomnia severity (ISI) without outliers** | | | | | |
| --- | --- | --- | --- | --- | --- |
|  | | | | | |
|  | **Baseline** | B | SE | Beta | t |
|  | (Constant) | -4.80 | 0.68 |  | -7.06*** |
|  | Sleep worry (APSQ) | 0.26 | 0.03 | 0.45 | 8.23*** |
|  | Dysfunctional beliefs (DBAS) | 0.13 | 0.17 | 0.04 | 0.44 |
|  | Pre sleep arousal (PSAS) | 0.17 | 0.02 | 0.35 | 7.52*** |
|  | Sleep safety behaviors (SRBQ) | 0.01 | 0.02 | 0.02 | 0.67 |
|  | R^2^_adj_ | 54.90% |  |  |  |
|  | *Note.* ** = *p < .01; *** = p < .001.* Baseline is based on *n* = 352 | | | | |

| Supplemental **Table S2 - Regression coefficients for the cross-sectional analyses, including sex, predicting insomnia severity (ISI)** | | | | | |
| --- | --- | --- | --- | --- | --- |
|  | | | | | |
|  | ***Baseline*** | *B* | *SE* | *Beta* | *t* |
|  | (Constant) | -4.42 | 0.91 |  | -4.89*** |
|  | Sex | -2.09 | 0.39 | -.02 | -0.53 |
|  | Sleep worry (APSQ) | 0.26 | 0.03 | 0.45 | 8.17*** |
|  | Dysfunctional beliefs (DBAS) | 0.14 | 0.17 | 0.04 | 0.84 |
|  | Pre sleep arousal (PSAS) | 0.18 | 0.02 | 0.35 | 7.47*** |
|  | Sleep safety behaviors (SRBQ) | 0.00 | 0.02 | 0.00 | 0.04 |
|  | R^2^_adj_ | 54.0% |  |  |  |
|  |  |  |  |  |  |
|  | **1-year later** | *B* | SE | Beta | t |
|  | (Constant) | -3.35 | 2.03 |  | -1.65 |
|  | Sex | -0.84 | 0.85 | -0.07 | -0.99 |
|  | Sleep worry (APSQ) | 0.25 | 0.07 | 0.42 | 3.41** |
|  | Dysfunctional beliefs (DBAS) | -0.49 | 0.39 | -0.15 | -1.26 |
|  | Pre sleep arousal (PSAS) | 0.24 | 0.05 | 0.49 | 5.06*** |
|  | Sleep safety behaviors (SRBQ) | 0.04 | 0.04 | 0.13 | 1.12 |
|  | R^2^_adj_ | 62.1% |  |  |  |
|  | *Note.* ** = *p < .01; *** = p < .001.* Baseline is based on *n* = 353 and 1-year follow-up on n = 79 | | | | |

| **Supplemental Table S3 - Regression coefficients predicting 1-year follow-up insomnia severity with baseline measures and sex** | | | | | |
| --- | --- | --- | --- | --- | --- |
|  | | | | | |
|  | **Step 1** | *B* | *SE* | *Beta* | *t* |
|  | (Constant) | 5.42 | 2.33 |  | 2.33* |
|  | Insomnia severity | 0.56 | 0.10 | 0.56 | 5.54*** |
|  | Sex | -1.12 | 1.21 | -0.09 | -0.92 |
|  | R^2^_adj_ | 30.0% |  |  |  |
|  |  |  |  |  |  |
|  | **Step 2** | *B* | *SE* | *Beta* | *t* |
|  | (Constant) | 7.35 | 3.12 |  | 2.37* |
|  | Sex | -1.62 | 1.23 | -0.13 | -1.33 |
|  | Sleep worry (APSQ) | -0.02 | 0.11 | -0.03 | -0.19 |
|  | Dysfunctional beliefs (DBAS) | -0.30 | 0.51 | -0.09 | -0.59 |
|  | Pre sleep arousal (PSAS) | -0.06 | 0.10 | -0.11 | -0.67 |
|  | Sleep safety behaviors (SRBQ) | 0.12 | 0.05 | 0.40 | 2.29* |
|  | Insomnia severity | 0.47 | 0.14 | 0.46 | 3.34** |
|  | R^2^_adj_ | 32.3% |  |  |  |
|  | *Note.* Regression model is based on *n* = 71  * = *p* < .05; ** = *p* < .01; *** *p* < .001 | | |  |  |
